# The therapeutic effect of Yinqiaosan decoction against influenza A virus infection by regulating T cell receptor signaling pathway

**DOI:** 10.1016/j.heliyon.2024.e36178

**Published:** 2024-08-13

**Authors:** Danting Li, Zekun Wang, Wenlei Wang, Zhihui Zheng, Hailin Wei, Qin Su, Mengmeng Yang, Yimeng Zhao, Xinyuan Zhang, Xiaocong Yu, Pinghu Zhang, Yachun Shu

**Affiliations:** a Department of Pharmacy, Affiliated Hospital of Nanjing University of Chinese Medicine & Jiangsu Province Hospital of Chinese Medicine, Nanjing, 210029, China; b Institute of Translational Medicine &Jiangsu Key Laboratory of Integrated Traditional Chinese and Western Medicine for Prevention and Treatment of Senile Diseases, Medical College, Yangzhou University, Yangzhou, 225009, China; c Jiangsu Province Seaside Rehabilitation Hospital, Lianyungang, 222042, China

**Keywords:** Yinqiaosan decoction, Influenza A, T cell receptor signaling pathway, PI3K, UHPLC-Q-TOF-MS/MS

## Abstract

**Background:**

Yinqiaosan decoction (YQSD), a traditional Chinese medicinal recipe, has been employed to treat influenza in China for approximately 300 years.

**Objective:**

Our study aimed to explore the mechanisms of YQSD against influenza via *in vivo* and *in vitro* experimental studies.

**Study design:**

and methods UHPLC-Q-TOF-MS/MS was utilized to examine the substances of the YQSD. The chemical components of YQSD detected by UHPLC-Q-TOF-MS/MS were used for network pharmacology analysis. The antiviral effect of YQSD *in vivo* was investigated. The potential mechanisms of YQSD in combating influenza, which were predicted from network pharmacology analysis, were validated *in vitro*.

**Results:**

By use of UHPLC-Q-TOF-MS/MS, 97 compounds were identified from YQSD. Network pharmacology analysis revealed that the therapeutic effect of YQSD against influenza may be associated with the regulation of T cell receptors (TCR) and Phosphoinositide 3-Kinase (PI3K)- protein kinase B (Akt) signaling pathways. Treatment with YQSD significantly prolonged the mean survival time of the mice and reduced lung injury due to the influenza A virus *in vivo*. It was discovered that YQSD efficiently inhibited the expression of inflammation-related cytokines. Moreover, YQSD has been found to significantly reduce the expression levels of cluster of differentiation 3 (CD3), monocyte chemoattractant protein-1 (MCP-1), and H1N1 virus nucleoprotein (NP), and prevent the decrease of epithelial cadherin (E-cadherin) protein. In addition, YQSD can inhibit the phosphorylation of the zeta chain of T cell receptor-associated protein kinase 70 (ZAP70) and PI3K proteins *in vitro*.

**Conclusion:**

The capacity of YQSD to suppress viral multiplication and inflammatory response by modulating T cell immunity may explain its effect against influenza viral pneumonia, which may involve the regulation of TCR and PI3K signaling pathways.

## Highlights

• YQSD can significantly reduce mice mortality resulting from the influenza A virus.
• YQSD attenuated influenza by reducing viral replication.
• YQSD can improve the occurrence of excessive inflammation *in vivo*.
• Regulating T-cell immunity may be a crucial mechanism of YQSD against influenza.

## Graphical abstract

**Figure:**
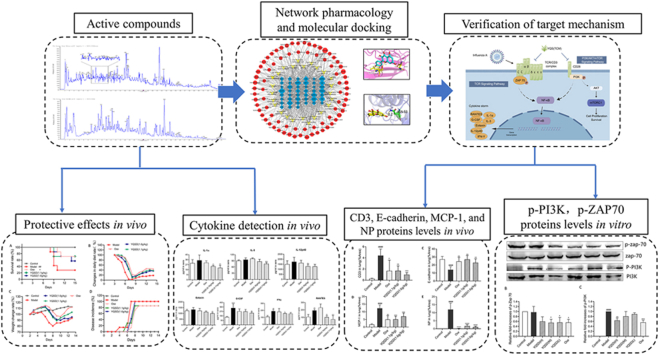

## Abbreviations

YQSD: Yinqiaosan decoction
Ose: oseltamivir
NP: H1N1 virus nucleoprotein
NA: neuraminidase
UHPLC-Q-TOF-MS/MS: Ultra High Performance Liquid Chromatography-Quadrupole-Time of Flight-Mass Spectrometry
TCID50: 50 % tissue culture infective dose
LD50: 50 % lethal dose
IAV: influenza A virus
E-cad: epithelial cadherin (E-cadherin)
CD3: cluster of differentiation 3
MCP-1: monocyte chemoattractant protein-1
RANTES: regulated upon activation normal T cell expressed and secreted
G-CSF: granulocyte colony stimulating factor Granulocyte colony stimulating factor
IL-12p40: interleukin 12 p40
IFN-γ: interferon-γ
IL-3: interleukin 3
IL-1α: interleukin 1α
PI3K: Phosphoinositide 3-Kinase
TCR: T cell receptor
ZAP70: zeta chain of T cell receptor associated protein kinase 70

## 1 Introduction

Influenza is currently considered one of the world's public health problems. Clinical epidemiological statistical analysis suggests that annual influenza A virus infections in 5 %–15 % of the global population result in 300 000 to 650 000 fatalities [1]. Previous research indicated that the influenza A virus (IAV) infection is the primary cause of serious lung damage and potential mortality due to the cytokine storm [2]. The cytokine storm induced by IAV results from the excessive activation of both innate and adaptive immune cells, involving macrophages, neutrophils, and effector T cells [3]. However, due to being short of effective therapeutic targets, effective strategies have not yet been found to suppress IAV-mediated hyperinflammation. It is rarely thought that T cell-mediated immune responses might worsen acute lung harm brought on by IAV infection given the advantage of IAV-specific CD4^+^ and CD8^+^ T cells to immunity for IAV infection. Recent studies indicate that IAV-mediated acute lung damage may be strongly influenced by T cell-mediated overactive immunological responses [4]. Furthermore, it has been reported that IAV-mediated acute lung injury could be effectively improved by inhibiting effector CD8^+^ T cell-mediated excessive inflammatory responses [5]. Therefore, it has been speculated that excessive inflammatory reactions caused by T cells may be a productive strategy for treating influenza viral lung injury.

In China, a traditional treatment recipe for influenza known as Yinqiaosan decoction (YQSD) has been in use for almost 300 years [6]. YQSD comprises 9 kinds of herbal medicine, including *Forsythia suspensa* (Thunb.) Vahl, *Lonicera japonica* Thunb., *Glycine max* (L.) Merr., *Platycodon grandiflorum* (Jacq.) A. DC., *Glycyrrhiza uralensis* Fisch, *Lophatherum gracile* Brongn., *Arctium lappa* L., *Mentha haplocalyx* Briq., *Schizonepeta tenuifolia* Eriq (Supplementary Material 1). Some clinical practices have shown that YQSD combined with antiviral Western medicine could markedly step up the efficiency of pneumonia due to viruses [7,8]. Research has demonstrated that YQSD could suppress the replication of IAV and inhibit excessive inflammatory responses [9]. However, the possible processes of YQSD for the therapy of influenza remained unclear. Considering that inhibiting T cell-mediated excessive immune response can effectively improve IAV-mediated lung injury, we attempt to investigate whether YQSD can exert pharmacological effects against IAV by regulating T cells. Therefore, we propose the following hypothesis, one of the potential pharmacological effects of YQSD in treating influenza may involve mitigating inflammatory responses by inhibiting T cell hyperactivation, thus protecting against influenza-induced lung injury. In this study, we employed network pharmacology to identify the immunoregulatory pathways through which YQSD treats influenza. Our research aimed to validate the hypothesis through *in vivo* and *in vitro* experiments, providing theoretical support for the effective clinical use of YQSD.

## 2 Materials and methods

### 2.1 YQSD preparation

The herbal medicines of YQSD were accurately weighed according to the composition ratio in Supplementary Material 1 (Table S1). The following herbs were soaked for 30 min, including *Forsythia suspensa* (Thunb.) Vahl, *Lonicera japonica* Thunb., *Glycine max* (L.) Merr., *Platycodon grandiflorum* (Jacq.) A. DC., *Glycyrrhiza uralensis* Fisch, *Lophatherum gracile* Brongn., and *Arctium lappa* L. Then the water with herbs was heated to boil, and *Mentha haplocalyx* Briq. and *Schizonepeta tenuifolia* Eriq. were added to boil for 5min. Then the solution was heated for 10 min and filtered out while it was hot. The solution was concentrated to 1 g/mL by rotary evaporator.

### 2.2 UHPLC-Q-TOF-MS/MS analysis

The YQSD solution, which was prepared by 2.1 with a concentration of 1 g/mL, was passed a 0.22 μm microporous membrane. The continuous filtrate obtained was the test solution. UHPLC-Q-TOF-MS/MS analysis was performed according to the chromatography and mass spectrometry conditions in Supplementary Material 1. In addition, the chemical compositions of YQSD were quantified by UHPLC in Supplementary Material 5.

### 2.3 Network pharmacological analysis

The potential effective ingredients of nine herbs in YQSD were collected by UHPLC-MS. The GeneCards database (https://www.genecards.org/) was searched for targets relevant to influenza. Intersection targets of components and disease were uploaded to the STRING database (https://cn.string-db.org/) to perform protein-protein interaction (PPI) analysis. Targets with a degree value greater than or equal to twice the average value were selected as core targets. These core targets were then imported into the Metascape database (http://metascape.org/) for Gene Ontology (GO) analysis and Kyoto Encyclopedia of Genes and Genomes (KEGG) pathway enrichment analysis. Visualization of the results was conducted using the bioinformatics platform (https://www.bioinformatics.com.cn/). Core targets, components, and the KEGG pathways were imported into Cytoscape 3.6.1 for topological analysis and construction of a component-target-pathway interaction network. AutoDock software has been used to dock the key components of YQSD with core targets. The results were visualized and processed through PyMOL software.

### 2.4 Virus and cell

The Dulbecco's modified Eagle's medium (DMEM) with 10 % fetal bovine serum, 100U/mL penicillin, and streptomycin were used to grow the Madin-Darby canine kidney (MDCK) cell, which was bought from the Type Culture Collection and cultured. Jurkat cell, a human T-lymphocyte leukemia cell, was kindly provided by Dr. Zhifa Wen (Nanjing Maternity and Child Health Care Hospital), and cultured in 1640 medium containing 10 % fetal bovine serum and 100 U/mL penicillin and streptomycin. Yangzhou University's Key Lab of Livestock and Poultry Infectious Diseases of the Ministry of Agriculture donated the influenza A virus, H1N1 (A/FM/1/47, mouse adaption strain). The 50 % tissue culture infective dose (TCID_50_) of the virus in MDCK cells and the LD_50_ of the virus in ICR mice were determined using the Reed-Munch technique. The Yangzhou University BSL-2 biosafety laboratory served as the site for all studies.

### 2.5 Anti H1N1 virus effect of YQSD *in vivo*

ICR mice (female, 14–15 g) came from Yangzhou University's Center for Comparative Medicine. The mice were kept in individual ventilated cages (IVC, Feng's, Suzhou, Jiangsu, China) at a temperature of 22 ± 2 °C during a 12-h light/dark cycle. The animal experiments conducted by Yangzhou University (202208006) were approved by the Ethics Committee. The experiments followed the guidelines of the Chinese Animal Protection Act and the National Research Council Criteria to ensure humane care for the animals.

The following is the LD_50_ virus detection method. The virus solution was diluted by a 10 fold series at 6 concentrations (1 × 10^−1^∼1 × 10^−6^). 36 ICR mice were divided into 6 groups, with 6 mice in each group at simple random. After anesthesia, each group of mice was intranasally inoculated with different concentrations of H1N1 virus once. Mice were continuously observed for 14 days to record their mortality status. The mortality rate was statistically analyzed and the LD_50_ of ICR mice was calculated.

An earlier approach was used to examine the protective effect of YQSD against infection with the H1N1 virus [10]. Preliminary experiments in ICR mice showed that when using YQSD at a clinical dose of more than 1.6 times, it showed mild toxic side effects (Supplementary Material 1). Therefore, the dosage of YQSD was determined to be 1.1 and 1.6 times the clinical dose in adults (60 kg). 60 mice were allocated into five groups at simple random: control group, model group, YQSD (1.6 g/kg) group, YQSD (1.1 g/kg) group, and oseltamivir (Ose) group (19.5 mg/kg). The control group served as the negative control, while the Ose group was the positive control. A suspension of the H1N1 virus at a sublethal dosage of 2 LD_50_ was intranasally administered once to the mice of the model group, YQSD group, and Ose group after they had been given anesthesia. After 48 h of infection, these mice were treated with oral YQSD (1.1 or 1.6 g/kg/day, the YQSD solution obtained from 2.1 was diluted with sterile water to the corresponding concentration), and Oseltamivir phosphate aqueous solution (19.5 mg/kg/day) for 5 days. Within the same number of days, the control and model groups were administered orally an equal volume of saline. 5 mice from each group were sacrificed on the 7th day after infection. Serum was isolated, and cytokines in serum were detected by high throughput liquid phase protein chip analysis (Bio-Plex Pro m Mouse Cytokine Standard 23-Plex 64313813, Group l, was purchased from Bio-Rad Laboratories Co., Ltd, California, USA). The lung tissues were weighted and then kept in formalin for immunohistochemical analysis and hematoxylin and eosin (H&E) staining. Daily records were made of any disease signs, changes in body weight, food variation, and mortality of the remaining mice (n = 7) in each group for 15 days. The effectiveness of protection was assessed based on the survival time and the rate of protection from death.

### 2.6 Immunohistochemical analysis

The expression levels of cluster of differentiation 3 (CD3), monocyte chemoattractant protein-1 (MCP-1), and H1N1 virus nucleoprotein (NP), and epithelial cadherin (E-cadherin) protein were detected through immunohistochemical analysis. First, seal the paraffin section and incubate it with 3 % BSA (69003435, Hushi Laboratory Equipment Co., Ltd, Shanghai, China) at room temperature for 10 min. Subsequently, the slices were incubated overnight with the primary antibody (detailed information can be found in Supplementary Material 1) at 4 °C. The next day, incubate the slide with secondary antibodies (KIT-5005, KIT-5002, Fuzhou Maixin Biotech. Co.,Ltd, China), and then perform DAB (DAB-1031, Fuzhou Maixin Biotech. Co.,Ltd, China) reaction for color development. Then observe the stained glass slide under a bright field microscope and capture the image. Quantitative analysis of positive staining areas was done using Image J software.

### 2.7 Preparation of medicated serum

Healthy male Sprague-Dawley rats (200–220 g) were provided by Sperford Biotechnology Co., Ltd. (Beijing, China). 27 SD rats were kept in individual ventilated cages (IVC, Feng's, Suzhou, Jiangsu, China) at a temperature of 22 ± 2 °C during a 12-h light/dark cycle. The daily dose of the drug tested on rats was converted from 8.5 times the clinical dose in adults (60 kg). This dose was obtained from preliminary experiments in Sprague-Dawley rats (Supplementary Material 1). The rats were simply randomly separated into 3 groups: the control group, the Ose group (21.42 mg/kg/day), and the YQSD group (8.28 g/kg/day) with 9 rats in each group. The rats had 3 days for adaptation, with a normal diet and drinking water, without medication treatment. The medicated serum of rats was collected as described [11]. Briefly, rats were fasted for 12 h and could drink freely before drug treatment. The rats were orally administered YQSD (8.28 g/kg/day, the YQSD solution obtained from 2.1 was diluted with sterile water to the corresponding concentration), oseltamivir phosphate granules aqueous solution (21.42 mg/kg/day), or an equal volume of saline for 5 days. Then rats were fasted 12 h before the last gavage (twice the dose). One hour after the last administration, the blood of the rats was collected after anesthetized. Collected blood was allowed to stand for 2 h for complete natural coagulation of red blood cells. It was then centrifuged at 3000 r · min^−1^ for 10 min to collect the supernatant, yielding the serum. The serum of the same group was combined, inactivated at 56 °C for 30 min, passed by 0.22 μm microporous membrane, and then kept on standby at −80 °C. The animal experiments conducted by Yangzhou University (YXYLL-2022-160) were approved by the Ethics Committee. The experiments followed the guidelines of the Chinese Animal Protection Act and the National Research Council Criteria to ensure humane care for the animals.

### 2.8 Cell viability determination

A stock solution of YQSD at a concentration of 1 g/mL was diluted with 1640 medium to a concentration of 25 600 μg/mL, which contained 2 % fetal bovine serum, 2 μg/mL 1-Tosylamido-2-phenylethyl chloromethyl ketone (TPCK) treated trypsin, and 100U/mL penicillin and streptomycin. A 0.22 μm microporous filtering membrane was used for sterilization of the diluted YQSD solution before it was further diluted to create 8 distinct testing concentrations (12 800, 6 400, 3 200, 1 600, 800, 400, 200, and 100 μg/mL). Cell viability after stimulation with YQSD was detected by the MTT method [10]. Jurkat cells were cultured in a 96-well plate with a cell concentration of 3 × 10^4^ cells per well until they reached 80 % confluency. For 48 h, Jurkat cells were exposed to YQSD at different concentrations (12 800, 6 400, 3 200, 1 600, 800, 400, 200, and 100 μg/mL). After removing the cell supernatant solution, the cells were given a dose of dimethyl sulfoxide (DMSO). The optical density of the samples was measured at 540 nm by a spectrophotometer.

YQSD or Ose medicated serum was diluted with 1640 medium (100U/mL penicillin and streptomycin), and the dilution concentration was 5 %, 10 %, and 20 %, which was filtered and sterilized through a 0.22 μm filter to obtain working fluid. The cell viability after stimulation with YQSD or Ose medicated serum on Jurkat cells for 24 h was determined by CCK8 assay [12].

### 2.9 Regulatory immune effects of YQSD on H1N1 virus infection *in vitro*

The TCID_50_ of H1N1 virus in cells was detected using the previously described method [13]. MDCK cells were seeded into a 96-well tissue culture plate with 2.5 × 10^4^ cells/well overnight. Influenza A virus was 10-fold diluted with DMEM for eight dilutions. Diluted viruses were inoculated into cell plates (four wells per dilution) for 4 days in a humidified CO_2_ incubator. MDCK cells were observed for cytopathic effects. The viral titer (log 10 TCID50/mL) was determined using the method described by Muench and Reed.

To investigate the antiviral mechanism of YQSD, the Jurkat cells infected with IAV were treated with YQSD, and the expression of related proteins was analyzed. The Jurkat cells suspension were transferred to a centrifuge tube, and its culture medium was discarded after centrifugated at 1, 000 r · min^−1^ for 10 min. As previously reported, Jurkat cells were infected with the virus of 100 times the 50 % tissue culture infection dose (100TCID_50_) for 1 h [10]. After being infected, the cells were resuspended in a 1640 medium with a 1/1000 dilution of 2 μg/mL TPCK trypsin and then moved to a 6-well plate. Jurkat cells were incubated with fresh 1640 medium that contained YQSD (200, 100, or 50 μg/mL) or Ose (10 μg/mL) for 24 h at a temperature of 35 °C and under a 5 % CO_2_ atmosphere.

To investigate how YQSD medicated serum affects immune cells, the Jurkat cells were treated with either YQSD or Ose medicated serum, and activated by CD3/cd28 antibody. The expression of phosphorylated zap70 proteins related to the TCR signaling pathway in Jurkat cells was measured. Jurkat cells were divided into the following groups based on the condition of treatment: blank group (1640 medium), blank serum group (10 % blank serum), CD3/CD28 antibody group (1640 medium and CD3/CD28 antibody), YQSD (L or H) serum group (5 % or 10 % YQSD medicated serum, and CD3/CD28 antibody), and Ose serum group (10 % Ose medicated serum and CD3/CD28 antibody). Blank and medicated serum was diluted with 1640 medium to concentrations of 5 % or 10 %. Jurkat cells were incubated with YQSD (5 % or 10 %), Ose (10 %) medicated serum, blank serum, or 1640 medium for 12 h and then were stimulated by CD3/CD28 antibody (20 μL/mL) for 30 min [14]. The blank group and blank serum group were not stimulated by CD3/CD28 antibody.

Western blot assay was used to detect the phosphorylation levels of zap70 and PI3K proteins as described previously, which was recorded in the supplementary material 1 [10].

### 2.10 Statistical analysis

Single-factor analysis of variance (ANOVA) or LSD-t-tests were also used to evaluate statistical differences across several groups. Analysis of the disparity in survival rates across groups was done using the Log-rank test. Statistical significance was defined as a *P*-value less than 0.05.

## 3 Results

### 3.1 Component analysis of YQSD by UHPLC-Q-TOF-MS/MS

**Fig. 1:**
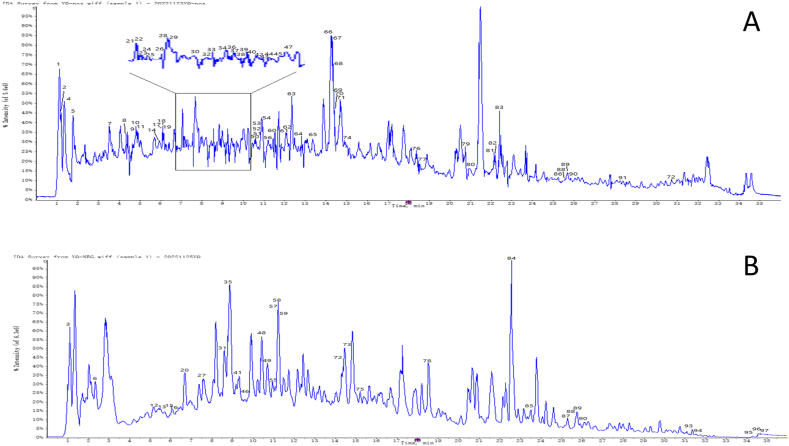
The total ion chromatogram of Yinqiaosan decoction (YQSD) was analyzed in both positive-ion mode (A) and negative-ion mode (B) by UHPLC-Q-TOF-MS/MS.

The total ion chromatogram of YQSD under positive and negative ionization modes was presented in Fig. 1 (A and B) and Supplementary Material 2. By comparing the retention time, precise molecular weight, and MS/MS fragment data with the local database, 97 chemicals were found.

### 3.2 Network pharmacological prediction results

**Fig. 2:**
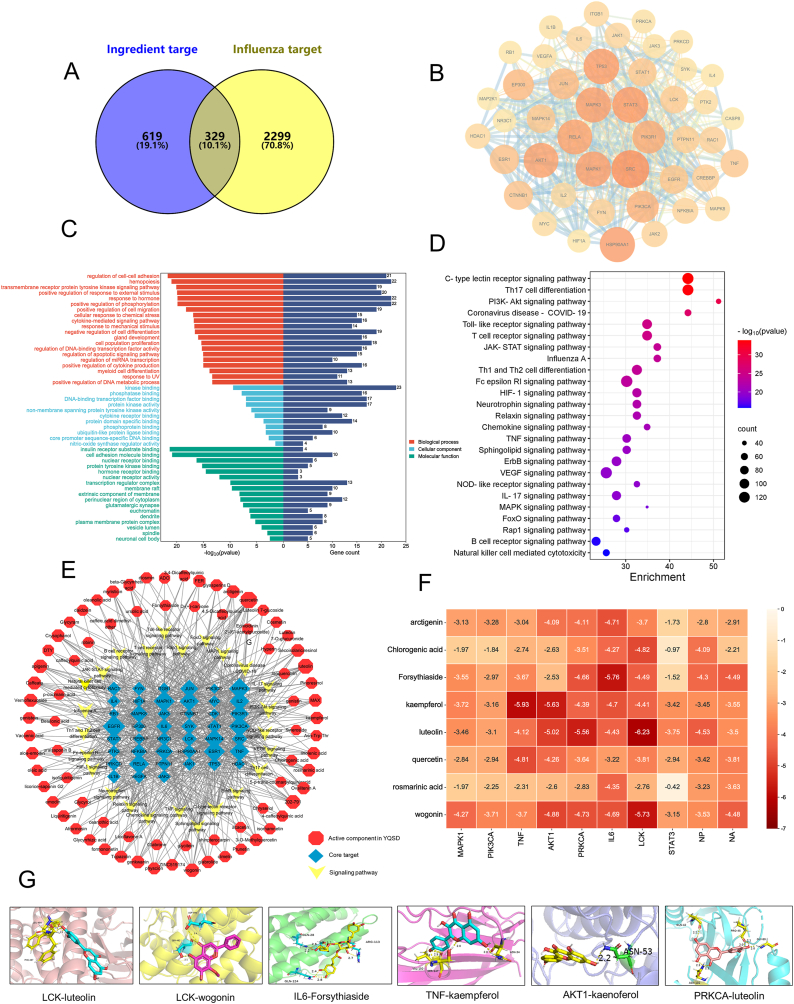
The network pharmacological prediction of Yinqiaosan decoction (YQSD). (A) The intersection target of YQSD and influenza. (B) PPI network of core targets. (C) GO enrichment analysis. (D) KEGG enrichment analysis. (E) Component-target-pathway network (red hexagon, active component in YQSD; blue diamond, core target; yellow fusiformis, signaling pathway). (F) Heatmap of molecular docking binding energy. (G) Molecular docking and binding sites.

There were 329 common targets related to YQSD and influenza (Fig. 2A). In the PPI network, 45 core targets were selected by twice the average degree value (Fig. 2B). The key potential targets, as shown in Table 1, were ranked in the top 20 by degree value in PPI analysis, such as MAPK1, MAPK3, AKT1, PIK3R1, PIK3CA, LCK and so on, which offering valuable references for subsequent mechanistic studies. Additionally, KEGG pathway enrichment analysis of influenza-related pathway sequencing was conducted, and the findings revealed that core target genes were predominantly enriched in pathways such as the PI3K-AKT and T cell receptor signaling pathways (Fig. 2C and D). The component-target-pathway network shows that each node represented a different component, target, or pathway, and the more connections between nodes, the higher the degree value. The top 5 components in terms of degree value were quercetin, luteolin, 202–791, apigenin, and wogonin (Fig. 2E). Based on the above results, active components of YQSD were selected for molecular docking with the H1N1 virus nucleoprotein (NP) and neuraminidase (NA), as well as top-ranked targets for the above results. The binding energy of both components and targets was less than 0 kJ mol^−1^, indicating that the key components in YQSD could effectively bind to the influenza-related targets (Fig. 2F). The results have been visualized in Fig. 2G. It was noteworthy that both PPI analysis and molecular docking results identify PIK3CA [15] and LCK [16] as potential targets, which were key targets of the PI3K-AKT and T cell receptor signaling pathways, respectively. Hence, based on the integrated network pharmacology findings, experimental validation of the aforementioned pathways was warranted.

**Table 1:** Top 20 important potential targets obtained from PPI analysis.

Gene Name	Target name	Degree
SRC	Proto-oncogene tyrosine-protein kinase Src	64
STAT3	Signal transducer and activator of transcription 3	59
TP53	Cellular tumor antigen p53	58
MAPK1	Mitogen-activated protein kinase 1	57
MAPK3	Mitogen-activated protein kinase 3	57
HSP90AA1	Heat shock protein HSP 90-alpha	57
RELA	Transcription factor p65	56
AKT1	RAC-alpha serine/threonine-protein kinase	56
PIK3R1	Phosphatidylinositol 3-kinase regulatory subunit alpha	51
PIK3CA	Phosphatidylinositol 4,5-bisphosphate 3-kinase catalytic subunit alpha isoform	48
EP300	Histone acetyltransferase p300	45
JUN	Transcription factor AP-1	45
ESR1	Estrogen receptor	41
CTNNB1	Catenin beta-1	40
EGFR	Epidermal growth factor receptor	39
MAPK14	Mitogen-activated protein kinase 14	39
STAT1	Signal transducer and activator of transcription 1-alpha/beta	38
TNF	Tumor necrosis factor	37
LCK	Tyrosine-protein kinase Lck	36
CREBBP	CREB-binding protein	35

### 3.3 The survival effect of YQSD on mice infected with H1N1 virus

**Fig. 3:**
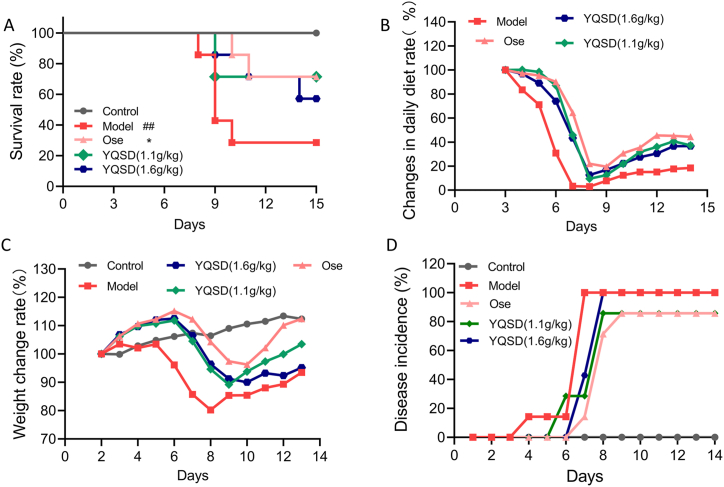
Anti-influenza virus effect of Yinqiaosan decoction (YQSD) or oseltamivir (Ose) *in vivo*. ICR mice exposed to the H1N1 virus were administered YQSD (1.1 g/kg or 1.6 g/kg), Ose (19.5 mg/kg), or an equivalent amount of saline orally for 5 days. The physical characteristics, weight, caloric consumption, and death of mice in each group were noted within 15 days (n = 7). (A) The percentage of infected mice who survived after receiving YQSD or Ose therapy. (B) After receiving therapy with YQSD or Ose, the daily diet rate of the mice with the infection changed. (C) The rate at which the treated infected mice's weight changed. (D) The illness prevalence in the treated infected mice. *P < 0.05, **P < 0.01, ***P < 0.001, ****P < 0.0001, *vs* model group; ^#^*P* < 0.05, ^##^*P* < 0.01, ^###^*P* < 0.001, ^####^*P* < 0.0001, *vs* control group.

ICR mice exposed to the H1N1 virus were administered YQSD (1.1 g/kg or 1.6 g/kg), Ose (19.5 mg/kg), or an equivalent amount of saline orally for 5 days. The physical characteristics, weight, caloric consumption, and death of mice in each group were noted within 15 days. The IAV-infected mice showed obvious clinical symptoms including unsmooth fur, inactivity, decreased intake of feed, decreased body weight, difficulty breathing, and even death, while after 48 h post-infection, YQSD treatment significantly alleviated the aforementioned signs of illness, prevented the dietary loss, and improved survival rate (Fig. 3A, B and C). As shown in Fig. 3D, the disease occurred in the YQSD (1.1 g/kg) group on the fifth day, which was later than that of the model group (on the third day), and the disease incidence (85 %) was relatively low. The body weight of the model group was about 30 % less than the initial body weight, and there was death between 8 and 10 days after infection. Moreover, compared with the model group (71.43 %), the death rate of the YQSD (1.1 g/kg) and (1.6 g/kg) groups (28.57 % and 42.86 %) was markedly reduced, and the mean survival time of the YQSD (1.6 g/kg) group (13.4 ± 2.4 days) was significantly prolonged (*P* = 0.0464), which was equivalent to Ose (13.7 ± 2.2 days) (Table 2). These results indicate that YQSD has a good therapeutic effect on H1N1 virus infection *in vivo.*

**Table 2:** The properties of Yinqiaosan decoction (YQSD) against H1N1 virus infection *in vivo* (n = 7).

Group	Dose	Count (n)	Death (n)	Death rate (%)	Death protective rate (%)	Average survival days	Life extension rate (%)
Control		7	0	0	/	15	
Model		7	5	71.43	/	10.7 ± 3.0	
Ose	19.5 mg/kg	7	2	28.57	60.00	13.7 ± 2.2 *	28.04
YQSD(L)	1.1 g/kg	7	2	28.57	60.00	13.3 ± 2.9	24.30
YQSD(H)	1.6 g/kg	7	3	42.86	40.00	13.4 ± 2.4 *	25.23

*P < 0.05, **P < 0.01, ***P < 0.001, ****P < 0.0001, *vs* model group.

### 3.4 The ability of YQSD to protect against acute lung damage brought on by the H1N1 virus

**Fig. 4:**
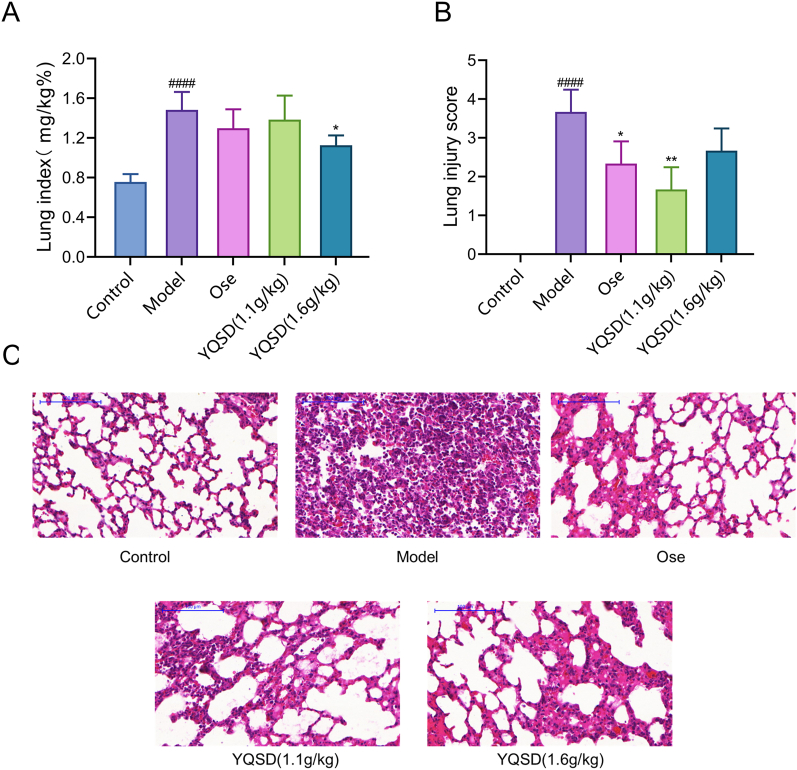
The properties of Yinqiaosan decoction (YQSD) or oseltamivir (Ose) on lung injury *in vivo* (n = 5). ICR mice that had been exposed to the H1N1 virus were administered YQSD (1.1 g/kg or 1.6 g/kg), Ose (19.5 mg/kg), or an equivalent amount of saline orally for 5 days. (A) The mice were sacrificed seven days after being infected. The lung tissue was gathered and weighed before the lung index was computed. (B) The histopathological score of lungs. A score of 0–4 represented normal, mild, severe, and very severe lung injury, respectively. Specifically, a score of 0 was given for normal lungs. 1 was given for mild interstitial pneumonia (less than 25 %). 2 was given for moderate interstitial pneumonia (25–50 %). 3 was given for severe interstitial pneumonia (50–75 %). 4 was given for very severe interstitial pneumonia (lung involvement greater than 75 %). (C) Representative histological alterations in the lung stained with hematoxylin and eosin (200x; scale, 20m). *P < 0.05, **P < 0.01, ***P < 0.001, ****P < 0.0001, *vs* model group; ^#^*P* < 0.05, ^##^*P* < 0.01, ^###^*P* < 0.001, ^####^*P* < 0.0001, *vs* control group.

ICR mice exposed to the H1N1 virus were administered YQSD (1.1 g/kg or 1.6 g/kg), Ose (19.5 mg/kg), or an equivalent amount of saline orally for 5 days. The mice were sacrificed 7 days after being infected. The lung tissue was gathered and weighed before the lung index (ratio of lung weight to body weight) was computed. Subsequently, the pathological changes of mouse lung tissue slices were observed using hematoxylin and eosin. The histopathological scores of the lungs were as follows [17]. A score of 0–4 represented normal, mild, severe, and very severe lung injury, respectively. Specifically, a score of 0 was given for normal lungs. 1 was given for mild interstitial pneumonia (less than 25 %). 2 was given for moderate interstitial pneumonia (25–50 %). 3 was given for severe interstitial pneumonia (50–75 %). 4 was given for very severe interstitial pneumonia (lung involvement greater than 75 %). As shown in Fig. 4A, the lung index of the YQSD (1.6 g/kg) group was considerably less than that of the model group (*P* = 0.0124). Furthermore, the model group also showed significant lung damage with widespread edema, severe cell necrosis, alveolar collapse or thickening, and severe infiltration of inflammatory cells. (Fig. 4C). However, YQSD treatment significantly improved the lung injury caused by IAV infection, which was similar to that of the Ose group. Moreover, the YQSD (1.6 g/kg) groups and Ose groups both had significantly lower lung damage ratings than the model group (Fig. 4B). These findings demonstrated that YQSD had a clear protective effect against acute lung damage brought on by the H1N1 virus.

### 3.5 Inhibitory effect of YQSD on excessive cytokine responses

**Fig. 5:**
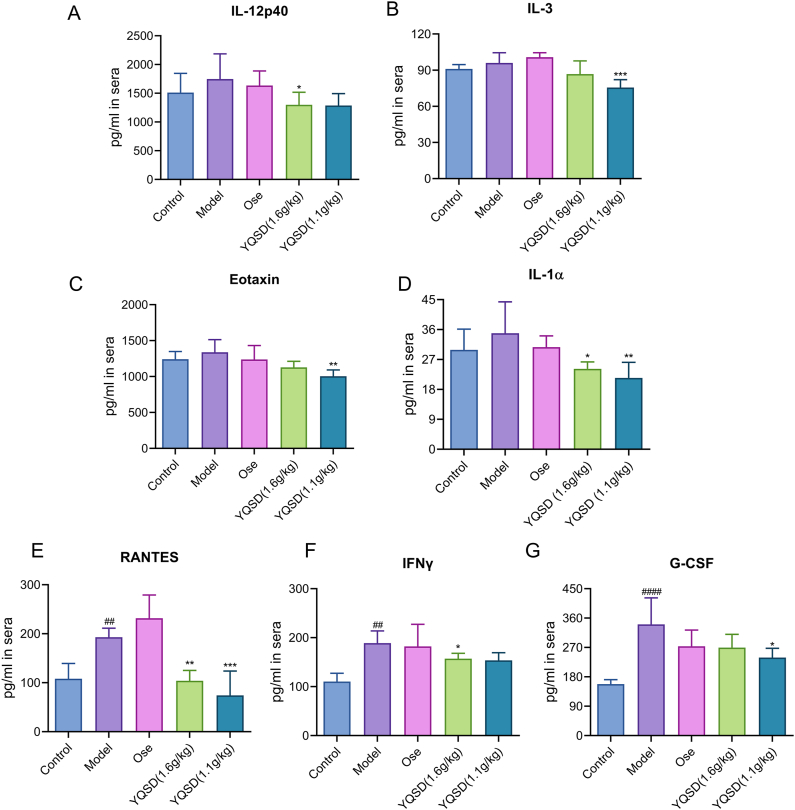
*In vivo* impact of Yinqiaosan decoction (YQSD) or oseltamivir (Ose) on H1N1-induced cytokines RANTES, G-CSF, eotaxin, IL-12p40, IL-3, IFN-γ, and IL-1α. Analyses of high throughput liquid phase protein chips were utilized to quantify the level of cytokines present in mouse serum (n = 5). *P < 0.05, **P < 0.01, ***P < 0.001, ****P < 0.0001, *vs* model group; ^#^*P* < 0.05, ^##^*P* < 0.01, ^###^*P* < 0.001, ^####^*P* < 0.0001, *vs* control group.

Analyses of high throughput liquid phase protein chips were utilized to quantify the level of cytokines present in mouse serum. As demonstrated in Fig. 5A–G, cytokines such as RANTES, G-CSF, eotaxin, IL-12p40, IL-3, IFN-γ, and IL-1α were considerably lower in the YQSD (1.1 or 1.6 g/kg) group when compared to the model group. These findings imply that YQSD therapy can effectively decrease pro-inflammatory factor expression produced by IAV *in vivo*. In addition, the effect of reducing pro-inflammatory factors of YQSD was better than Ose, positive control.

### 3.6 Mechanism of YQSD in treating influenza A virus infection *in vivo*

**Fig. 6:**
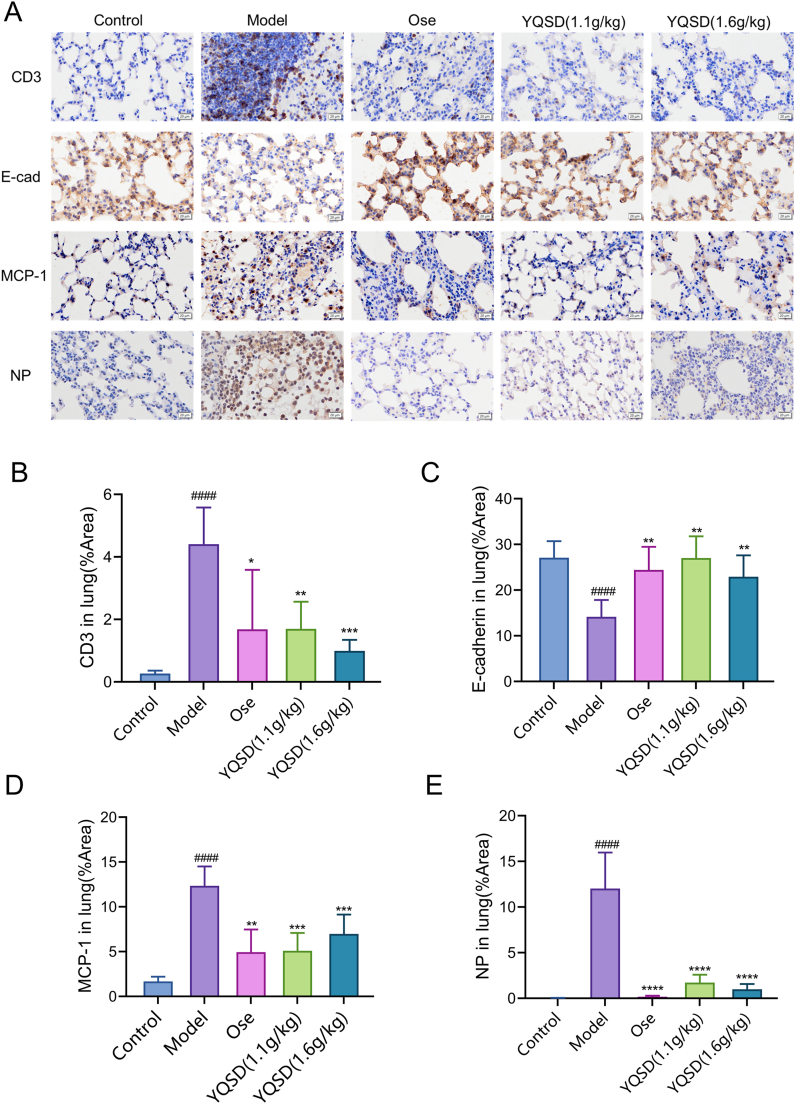
Effect of Yinqiaosan decoction (YQSD) or oseltamivir (Ose) on the expression of E-cadherin (E-cad), cluster of differentiation 3 (CD3), H1N1 virus nucleoprotein (NP), and monocyte chemoattractant protein-1 (MCP-1) proteins in mouse lung tissue. (n = 3) (A) Immunohistochemical analysis in mouse lungs. Magnification × 200. (B–E) The value for average density. Three distinct tissues of mice per group, and three randomly chosen regions from each slide. *P < 0.05, **P < 0.01, ***P < 0.001, ****P < 0.0001, *vs* model group; ^#^*P* < 0.05, ^##^*P* < 0.01, ^###^*P* < 0.001, ^####^*P* < 0.0001, *vs* control group.

Using image J software, three regions from different tissues of each group of mice were randomly selected for positive mean density analysis (n = 3). The results of immunohistochemistry analysis revealed that in contrast to the lung tissue of the model group, which had higher brown staining with CD3, MCP-1, and NP antibodies, as shown in Fig. 6A–E, the lung tissue of the YQSD group only displayed faint positive, which was a significant difference from the model group (*P* < 0.05 or 0.01). Additionally, the lung structures appeared to be intact since E-cadherin expression levels were much higher in the YQSD or Ose group than in the model group (strong positive staining with anti-E-cadherin antibody) (*P* < 0.01) (Fig. 6C). These findings suggested that the ameliorative effect of YQSD on influenza viral lung injury may be associated with T cell infiltration and inflammatory injury. In addition, NP helps to stabilize the viral genome and regulate synthesis and replication transcription [18]. YQSD can reduce the expression level of NP, indicating that YQSD can directly inhibit the replication of influenza A virus.

### 3.7 YQSD inhibits TCR and PI3K signal pathway *in vitro*

**Fig. 7:**
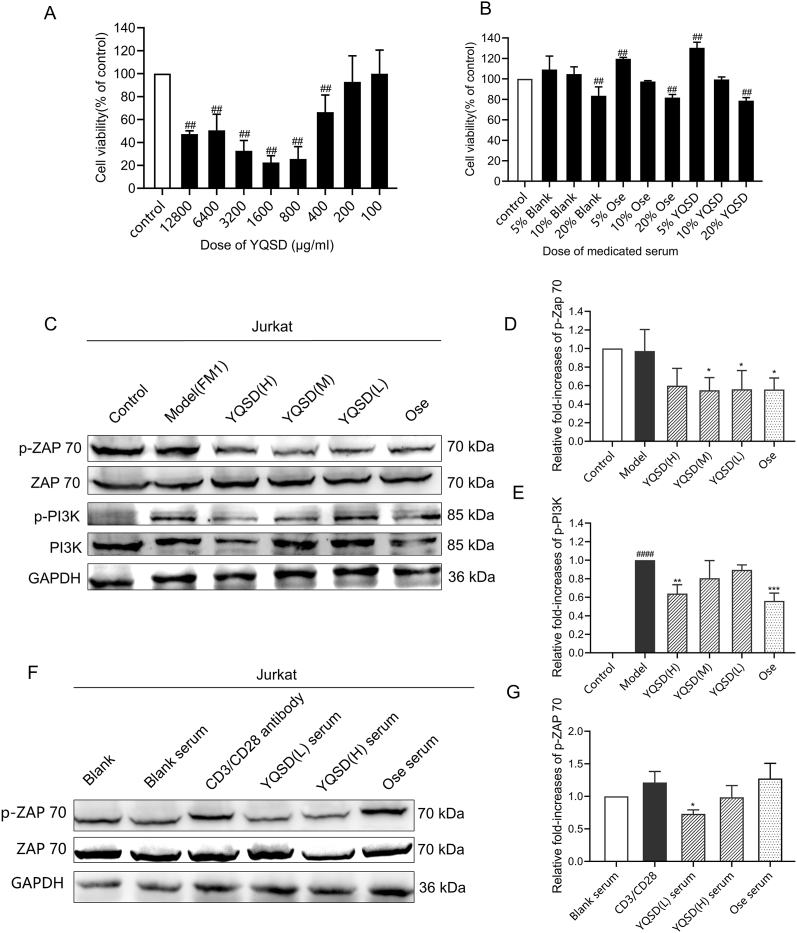
The effect of Yinqiaosan decoction (YQSD) or various medicated sera at different doses on cell viability. The effect of YQSD or oseltamivir (Ose) on T cell receptors (TCR) and PI3K signal pathway. (A) After YQSD (1280, 6400, 3200, 1600, 800, 400, 200, and 100 μg/mL) was applied to Jurkat cells for 48 h, the cell viability was assessed using the MTT technique (n = 3). (B) Jurkat cells were stimulation with various medicated sera (5 %, 10 %, and 20 %) for 24 h. Cell viability was discovered using the CCK8 technique (n = 3). (C) Jurkat cells were treated with YQSD (H: 200 μg/mL, M: 100 μg/mL, L: 50 μg/mL) or Ose (10 μg/mL) for 24 h after being infected with the H1N1 virus, and the expression levels of p-ZAP70 and p-PI3K were evaluated by western blot. (D, E) The relative expression level of p-ZAP70 and p-PI3K (n = 3). *P < 0.05, **P < 0.01, ***P < 0.001, ****P < 0.0001, *vs* model group; ^#^*P* < 0.05, ^##^*P* < 0.01, ^###^*P* < 0.001, ^####^*P* < 0.0001, *vs* control group. (F) After being treated with YQSD (H: 10 %, L: 5 %) and Ose (10 %) medicated serum for 12 h, Jurkat cells were stimulated by CD3/CD28 antibody (20 μL/mL) for 30 min, and the expression level of p-ZAP70 was evaluated by Western blot. (G) The relative expression levels of p-ZAP70 (n = 3). *P < 0.05, compared with the CD3/CD28 antibody group; #*P* < 0.05, compared with the blank serum group.

MTT and CCK8 assays were used to assess cell viability. At 200 μg/mL of YQSD and 10 % medicated serum, no cytotoxicity was seen in Jurkat cells (Fig. 7A and B). Decoction or medicated serum's antiviral properties were therefore tested at doses that had little to no cytotoxicity in Jurkat cells. When applied to Jurkat cells that had been exposed to the H1N1 virus, YQSD therapy reduced the phosphorylation of ZAP70 and PI3K in comparison to the model group (*P* < 0.05 or 0.01) (Fig. 7C, D and E). Similar to this, treatment with Ose decreased ZAP70 and PI3K phosphorylation. Moreover, a significant reduction of the phosphorylation level of ZAP70 in Jurkat cells treated with YQSD (5 %) medicated serum was observed, compared with the CD3/CD28 antibody group (*P* < 0.05) (Fig. 7F and G). The row western blot data were Supplementary Material 3. These results show that YQSD may be possible to successfully decrease the overactive immune system by inhibiting the TCR and PI3K signal pathways.

## 4 Discussion

IAV primarily affects pulmonary epithelial cells and alveolar macrophages, but these target cells may release inflammatory mediators, which may attract a lot of inflammatory cells, including neutrophils, effector T cells, and macrophages, leading to lung damage [19]. For example, extensive immune cell infiltration, particularly T cell infiltration, is not particularly advantageous in the advanced stages of IAV infection. Lung damage might be brought on by overactive immunological responses mediated by T cells [20]. Studies have reported that IFN-γ, MCP-1, and MIP-2 derived from CD8 T cells is an important contributor to IAV-induced lung injury [21,22]. More seriously, overactivated T cells can directly mediate cytokine storms in H1N1 virus infection [23]. Therefore, inhibiting the overactivated T cell effect may be a pathway to reduce lung injury caused by IAV.

In China, some traditional decoctions such as Guizhi and Mahuang decoction, Xinjiaxiangruyin, and YQSD had therapeutic effects on influenza, while previous researchers have suggested that YQSD had a good anti-influenza effect and was more suitable for heat-infected lung influenza and inflammation [24]. Additionally, YQSD was regarded as one of the most often prescribed medications for the therapy of infections of the upper respiratory system [25], but its potential mechanism against IAV remains unclear. Consistent with the reported research, our results also confirmed that YQSD was effective for treating IAV infection *in vivo* even if administered 48 h after infection. Moreover, YQSD treatment could significantly improve lung tissue pathology, which has been confirmed by pathological and immunohistochemical analysis. Additionally, IAV infection-induced expression of RANTES, G-CSF, eotaxin, IL-12p40, IL-3, IFN-γ, and IL-1 was significantly suppressed by YQSD therapy. The nucleoprotein (NP) encapsulates the viral RNA genome to form a ribonucleoprotein (RNP) complex, stabilizing the genome and regulating its synthesis and replication transcription [18]. Immunohistochemical analysis results indicated that YQSD may impede influenza by disrupting NP synthesis in the viral replication cycle. E-cadherin is a key component of adhesive bonding that contributes to the integrity of the pulmonary epithelial barrier [26]. The shedding of E-cadherin leads to increased epithelial permeability and lung injury [27]. This study demonstrates that E-cadherin expressions in mice lung tissues significantly decreased post-virus infection, signifying lung injury. However, E-cadherin levels were higher in YQSD-treated, virus-infected mice, suggesting the protective role of YQSD against H1N1-induced lung injury by preserving E-cadherin integrity. In addition, T lymphocytes play a crucial role in the immune system, and the CD3 molecule is conventionally used to quantify inflammation-associated infiltration of T cells [28]. It was noted that the levels of expression of CD3 and inflammatory mediator MCP-1 in the tissue of the lungs of the YQSD treatment group were noticeably decreased, which suggests that the beneficial impact of YQSD on influenza viral pneumonia may be related to the inhibition of CD8^+^ T cell-mediated excessive immune response induced by IAV. Additionally, studies have suggested that upon pharmacological blockade of effector T cell activation, proliferation, and accumulation in the lungs following influenza infection, there was a downregulation of proinflammatory cytokines such as IL-1 and MCP-1 [5,29]. This confirms that excessive inflammation caused by influenza can be mitigated by inhibiting T cell involvement, presenting a new therapeutic approach for treating cytokine storms induced by influenza. Additionally, treatment administered 48 h after infection demonstrated that YQSD can prevent aberrant T cell activation and the production of many inflammatory factors.

The network pharmacology was utilized to forecast the probable targets to better clarify the possible effective ingredients and mechanisms of action of YQSD in treating influenza. Results suggested that the potential components and targets of YQSD were complex. After comprehensive reference to the ranking of components in the component-target-pathway network and molecular docking results, nine potential active constituents were identified, including quercetin, luteolin, baicalein, arctigenin, rosmarinic acid, kaempferol, glycyrrhizin, chlorogenic acid, and forsythoside A. Among these, quercetin [30], luteolin [31], chlorogenic acid [32], and forsythoside A [33] have been reported to possess anti-influenza properties in the literature. In addition, our findings suggested that the TCR and PI3K signaling pathways, which were closely associated with immune modulation and were among these signaling pathways, may be crucial to the therapeutic action of YQSD against influenza. On the one hand, the TCR signaling pathway activates T cells by promoting the phosphorylation of ζ Chain related 70 kDa protein (ZAP70) [34]. On the other hand, the phosphorylation of ZAP70 promotes T cell proliferation and activates the downstream NF-κB signaling pathway to induce excessive inflammatory responses [35]. Moreover, The PI3K signaling pathway is also activated by TCR signaling, but the PI3K/Akt/mTOR signaling pathway promotes T cell growth and inhibits cell death and autophagy [36]. The results from network pharmacology suggested that controlling these above two pathways might be an effective strategy to inhibit T cell-mediated excessive inflammation. Combined with preliminary *in vivo* experiments exploring its mechanism, it was found that YQSD can reduce the expression of CD3 and MCP-1, indicating T-cell infiltration and inflammatory responses in mouse lung tissue. We were intrigued by this and decided to further investigate the mechanism of T cells, also referring to network pharmacology predictions to validate pathways related to T cells. We employed Jurkat cells as a model *in vitro* for biological validation to confirm the aforementioned conjecture. Compared with the CD3/CD28 antibody group the phosphorylation level of ZAP70 in Jurkat cells incubated with CD3/CD28 antibody was significantly reduced in YQSD medicated serum group, suggesting that YQSD treatment could suppress the activation of T cells. Moreover, in Jurkat cells infected by the H1N1 virus, our results also confirmed that YQSD treatment could inhibit the phosphorylation of ZAP70 and PI3K, further confirming the above conclusion. Further evidence that YQSD therapy significantly reduced the production of RANTES, G-CSF, eotaxin, IL-12p40, IL-3, IFN-γ, and IL-1 revealed that YQSD's potential mechanism of treating influenza virus pneumonia may include suppressing the "cytokines storm". Our final results confirm that YQSD may protect against IAV-mediated lung harm by repressing TCR and PI3K signaling pathways, which can reduce T cell proliferation and promote overactive T cell death and autophagy thus decreasing the creation of inflammatory factors and target cell injury. This discovery provides theoretical support for early influenza control and the prevention of a "cytokine storm", offering a reference for the clinical application of YQSD. While YQSD had therapeutic effects on influenza, its potential side effects or toxicity were also worth considering. The literature indicates that *Lonicera japonica* Thunb., a component of YQSD, can produce certain toxicity after extensive or prolonged oral administration [37]. We hypothesized that this may be related to the immunosuppressive effects of YQSD, as prolonged suppression of the immune system increases the risk of infection by other pathogens. Therefore, avoiding excessive and prolonged use of YQSD can enhance its safety in treating influenza.

In this study, our results indicated that treatment with YQSD effectively improves lung injury and lengthens the mean number of days that sick mice survive. The main components of YQSD have been identified and used for network pharmacological analysis. Ulteriorly, the results of network pharmacological revealed that the potential mechanisms of YQSD against IAV may associate with TCR and PI3K-Akt signaling pathways. Moreover, the above mechanisms of YQSD have been validated *in vitro* and *in vivo* biological experiments. Our findings provide compelling support for the use of YQSD in IAV infection therapeutic therapy in the future. However, it was necessary to acknowledge the limitations of this work. This study only preliminarily demonstrated the mechanism of action on immune cells *in vitro*, which was a limitation of the experimental model. In our future work, we intend to conduct in-depth validation of the mechanism of action discovered in this study through *in vivo* experiments.

## 5 Conclusion

**Fig. 8:**
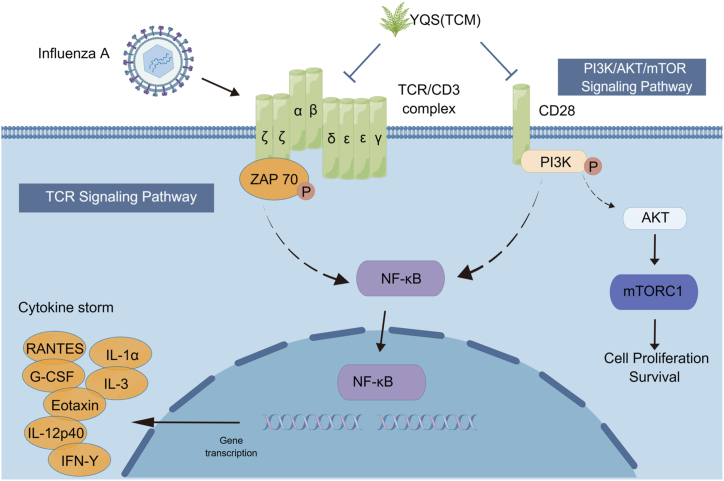
Potential model of Yinqiaosan decoction (YQSD) regulating the T cell receptors (TCR) and PI3K signaling pathway. By Figdraw.

In conclusion, our findings show that the *in vivo* therapeutic benefit of YQSD against IAV-induced acute lung damage may be connected to inhibiting viral multiplication and inflammatory response through modulating T cell immunological response. *In vitro*, YQSD may dramatically decrease the phosphorylation of ZAP70 and PI3K proteins, which may be connected to TCR and PI3K signaling pathway regulation (Fig. 8).

## Ethical approval statement

The Experimental Animal Ethics Committee of Yangzhou University and the Ethics Committee of Yangzhou University School of Medicine approved all animal experiments conducted (NO. 202208006 and NO. YXYLL-2022-160) and formulated animal humane care following the guidelines of the Chinese Animal Protection Act and the National Research Council Criteria.

## Funding

This work was financially supported by the Project of Natural Science Foundation of Nanjing University of Chinese Medicine, Jiangsu Provence, China (grant numbers XZR2020020), Nanjing Intellectual Property Plan Project, Jiangsu Provence, China (grant numbers GJ20231160_01), the Postgraduate Research & Practice Innovation Program of Jiangsu Province, China (grant numbers SJCX22_0759) and the 10.13039/501100002949Jiangsu Province Traditional Chinese Medicine 10.13039/100006180Technology Development Plan Project, China (grant numbers MS2022155).

## Data availability statement

Data will be made available on request.

## CRediT authorship contribution statement

**Danting Li:** Writing – original draft, Software, Methodology, Investigation, Formal analysis, Conceptualization. **Zekun Wang:** Visualization, Software, Investigation, Formal analysis. **Wenlei Wang:** Investigation, Formal analysis. **Zhihui Zheng:** Writing – review & editing, Investigation. **Hailin Wei:** Investigation. **Qin Su:** Investigation. **Mengmeng Yang:** Investigation. **Yimeng Zhao:** Investigation. **Xinyuan Zhang:** Investigation. **Xiaocong Yu:** Investigation. **Pinghu Zhang:** Writing – review & editing, Supervision, Resources, Methodology. **Yachun Shu:** Writing – review & editing, Supervision, Project administration, Funding acquisition, Conceptualization.

## Declaration of competing interest

The authors declare that they have no known competing financial interests or personal relationships that could have appeared to influence the work reported in this paper.

## Footnotes

Appendix A Supplementary data to this article can be found online at https://doi.org/10.1016/j.heliyon.2024.e36178.
