# Supplementary material for: The therapeutic effect of Yinqiaosan decoction against influenza A virus infection by regulating T cell receptor signaling pathway
Source: Heliyon. 2024 Aug 13;10(16):e36178. doi: 10.1016/j.heliyon.2024.e36178 (PMC11382312; doi:10.1016/j.heliyon.2024.e36178)
Supplement: Multimedia component 1 [file mmc1.docx]

**Materials and methods**

**1 Reagent**

The herbs of YQSD were provided by Jiangsu Province Hospital of Chinese Medicine, Nanjing City, Jiangsu Province, China. The detailed information of each herb was displayed in Table S1. Dulbecco’s modified Eagle’s medium (DMEM) (KG500061860) and RPMI-1640 medium (KG500025936) were obtained from Jiangsu keygen Biotechnology Co., Ltd. (Jiangsu, China). Trypsin 1:250 was obtained from Solarbio (Beijing, China). Fetal bovine serum (11011-8611) was obtained from Zhejiang Tianhang Biotechnology Co., Ltd (Zhejiang, China). CD3/CD28 antibody (100079289) was purchased from stem cell (Canada). The thiazolyl blue tetrazolium bromide (MTT)(ST316) was purchased from Beyotime Biotechnology (Shanghai, China). The Cell Counting Kit-8 (CCK-8) (K1018) was purchased from ApexBio Technology (Houston, USA). The primary antibodies of phosphorylated ZAP70 (p-ZAP70) (2717), ZAP70 (3165), phosphorylated PI3K (p-PI3K) (4228) and PI3K (4257) was purchased from Cell Signaling Technology (USA). The primary antibody of CD3 (Ab16669) and NP (Ab20343) was purchased from Abcam Company (UK). The primary antibody of E-cadherin (20874-1-AP) was purchased from Proteintech Group，Inc. (Wuhan, China). The primary antibody of MCP-1 (DF7577) was purchased from Affinity Biosciences (Jiangsu, China). Oseltamivir phosphate (S2597) was purchased from Selleck Chemical Co., Ltd. (Shanghai, China). Oseltamivir phosphate granules (6002207058) were purchased from East Sunshine Changjiang Pharmaceutical Co., Ltd. (Hubei, China). Bio-Plex Pro m Mouse Cytokine Standard 23-Plex, Group l (64313813) was purchased from Bio-Rad Laboratories Co., Ltd. (California, USA).

| Name | Medicinal parts | Composition ratio | Source | Lot number | Place of production |
| --- | --- | --- | --- | --- | --- |
| *Forsythia suspensa* (Thunb.) Vahl | fruit | 9 | Anhui Ma'anshan Jingquan Traditional Chinese Medicine Slices Co., Ltd., China | 220301 | Henan, China |
| *Lonicera japonica* Thunb. | bud or with blooming flowers | 9 | Anhui Ma'anshan Jingquan Traditional Chinese Medicine Slices Co., Ltd., China | 220501 | Shandong, China |
| *Glycine max* (L. ) Merr. | Fermented processed products of mature seeds | 5 | Anhui Wansheng Traditional Chinese Medicine Slices Co., Ltd., China | 220301 | Anhui, China |
| *Platycodon grandiflorum* (Jacq.) A. DC. | root | 6 | Anhui Ma'anshan Jingquan Traditional Chinese Medicine Slices Co., Ltd., China | 220402 | Anhui, China |
| *Glycyrrhiza uralensis* Fisch | roots and rhizomes | 5 | Anhui Ma'anshan Jingquan Traditional Chinese Medicine Slices Co., Ltd., China | 220401 | Gansu, China |
| *Lophatherum gracile* Brongn. | stem and leaf | 4 | Anhui Xintai Pharmaceutical Co., Ltd., China | 220201 | Ya'an, Sichuan, China |
| *Arctium lappa* L. | ripe fruit | 9 | Anhui Xintai Pharmaceutical Co., Ltd., China | 211101 | Baoding, Hebei, China |
| *Mentha haplocalyx* Briq. | aboveground part | 6 | Anhui Xiehecheng Pharmaceutical Co., Ltd., China | 21082004 | Fuyang, Anhui, China |
| *Schizonepeta tenuifolia* Eriq. | aboveground part | 5 | Anhui Wansheng Traditional Chinese Medicine Slices Co., Ltd., China | 220501 | Hebei, China |

Table S1. Basic information on the composition of medicinal herbs in Yinqiaosan decoction (YQSD).

**2** **UHPLC-Q-TOF-MS/MS analysis**

UHPLC-Q-TOF-MS/MS system was composed of ExionLC ultra-high performance liquid chromatograph (AB SCIEX, USA) and Q-Tof 5600^+^ high-resolution mass spectrometer (AB SCIEX, USA). A C18 column ACQUITY UPLC^®^ HSS T3 column (2.1×100 mm, 1.8 μm) was used for chromatographic separations. The chromatographic column was adjusted at a flow rate of 0.2 mL/min, a temperature of 40 ℃, and an injection volume of 2 μL. Mobile phase A was composed of 0.05% formic acid aqueous solution, and mobile phase B was composed of 100% acetonitrile. The gradient elution procedure was as follows，0～16 min，10%～35%(B)；16～18 min，35%～35%(B)；18～28 min，35%～60%(B)；28～32 min，60%～100%(B)；32～34 min，100%～10%(B)；34～36 min，10%～10%(B). An electrospray ionization source was utilized in both positive and negative ion modes. The measurement parameters were as follows: ionization temperature, 550.0 ℃, atomizing gas, 55 psi, auxiliary heating gas, 55 psi, curtain gas, 30 psi. The collection scope of primary and secondary mass spectrometry was m/z 50~1500, declustering potential, 60 V, collision Energy, 45 eV.

**3** **Previous experiments in ICR mice**

ICR mice (female, 14-15 g) came from Yangzhou University's Center for Comparative Medicine. The mice were kept in Individual Ventilated Cages (IVC, Feng's, Suzhou, Jiangsu, China) at a temperature of 22 ± 2 °C during a 12-hour light/dark cycle. The animal experiments conducted by Yangzhou University (202208006) were approved by the Ethics Committee. The experiments followed the guidelines of the Chinese Animal Protection Act and the National Research Council Criteria to ensure humane care for the animals.

YQSD was prepared according to the method described in section 2.1 of the main text and diluted with sterile water to the appropriate concentration. ICR mice were allocated into five groups at simple random: control group, model group, YQSD (3.3 g/kg, 3.3 times the clinical dose for adults) group. A suspension of the H1N1 virus at a sublethal dosage of 2 LD50 was intranasally administered once to the mice of the model group and YQSD group after they had been given anesthesia. After 48 hours of infection, these mice were treated with oral YQSD (3.3 g/kg/day) for 5 days. Within the same number of days, the control and model groups were administered an equal volume of saline. Daily records were made of any disease signs, changes in body weight, food variation, and mortality of the mice (n=7) in each group for 15 days. The result showed that the mortality rate of mice was 71.43%, indicating that YQSD had slight toxic side effects at this dose.

**4 Previous experiments in** **Sprague-Dawley rats**

As our team has previously published experimental reports, the anti-inflammatory effects of YQSD on Sprague-Dawley rates have been studied[1]: In short, YQSD was prepared according to the main text 2.1, and then diluted to the desired concentration with sterile water. Male Sprague Dawley rats were divided into model group, YQSD (5g/kg/d) group, and YQSD (10g/kg/d) group, with 10 mice in each group. Each group of rats was intraperitoneally injected with LPS with a body weight of 100 μg/kg for modeling. After 1 hour of LPS injection, YQSD group rats were orally given of YQSD sterile water dilution solution, while the model group was orally with sterile 0.9% sodium chloride solution. Administration lasts for 7 days. At 1, 2, 3, 4, 5, 6, and 7 hours after LPS injection, rectal temperature (Ti) was measured and the temperature change value Δ T (Δ T=Ti-T0) was calculated. The results showed that the dose range of 5-10g/kg/d had antipyretic and anti-inflammatory effects, and can be used for the preparation of medicated serum in the main text.

**5** **Western blot analysis**

For Western blot analysis, Jurkat cells were lysed using RIPA lysis buffer (Beyotime, China) and then boiled at a temperature of 100 °C for 10 minutes. The protein samples were separated using 10% or 12% SDS-PAGE gels and then transferred onto a nitrocellulose (NC) membrane with a pore size of 0.45 μm from Millipore. The membranes were sealed in a TBST solution containing 5% skim milk for 1 hour. After that, they were incubated with primary antibodies against p-ZAP, ZAP, p-PI3K, PI3K, and GAPDH (as a control) for 12 hours at a temperature of 4°C. Finally, the membranes were incubated with secondary antibodies that were conjugated with horseradish peroxidase for 1 hour at room temperature. The membranes were examined using an enhanced chemiluminescence kit (Millipore, Billerica, MA, USA) and a Molecular Imager SH-523 System (Hangzhou Shenhua, Hangzhou, China). To determine the relative amount compared to GAPDH, densitometric analysis was conducted using SHST analysis software (Shenhua, Hangzhou, China).

**References**

[1] S. Yachun, Z. Xuanxuan, C. Yajun, Q. Kunming, C. Baochang, Study on the spectrum effect relationship of anti-inflammatory activity of yinqiao san decoction at different post treatment times, China dournal of Traditional chinese Medicine and Pharmacy 33 (09) (2018) 4161-4166,

https://kns.cnki.net/kcms2/article/abstract?v=jkwd3qsBIELYJQE-IllxH4dTUyh6rxhsnyuWCY2PH5THNZT4xjEjotnI68DIGEJuIvUrzl7wLX2eYLUreh1-SDNMCDk55gTp0typHl2AOJs-Q_dUpEC8hT3jrOawMmKQYGGMa4tX8Ujehk8raF84Y8o5l6MgQ6YD&uniplatform=NZKPT&language=CHS.
