# Supplementary material for: The therapeutic effect of Yinqiaosan decoction against influenza A virus infection by regulating T cell receptor signaling pathway
Source: Heliyon. 2024 Aug 13;10(16):e36178. doi: 10.1016/j.heliyon.2024.e36178 (PMC11382312; doi:10.1016/j.heliyon.2024.e36178)
Supplement: Multimedia component 3 [file mmc3.docx]

Figure S4A-p-ZAP 70 Figure S4B- ZAP 70





Figure S4C-GAPDH

Figure S4 Originals blots corresponding to Fig7F.
