# Supplementary material for: The therapeutic effect of Yinqiaosan decoction against influenza A virus infection by regulating T cell receptor signaling pathway
Source: Heliyon. 2024 Aug 13;10(16):e36178. doi: 10.1016/j.heliyon.2024.e36178 (PMC11382312; doi:10.1016/j.heliyon.2024.e36178)
Supplement: Multimedia component 4 [file mmc4.docx]

**Quantitative analysis of YQSD through UHPLC**

The components of Yinqiaosan docotion (YQSD) were quantitatively detected through Ultra High Performance Liquid Chromatography (UHPLC).

1. Materials and methods

1.1 Reagent

Chlorogenic acid (110753-202119), rutin (10080-202012), hesperidin (110721-202220), arctiin (110819-201812), phillyrin (110821-200711) (purity ≥98%) were purchased from National Institutes for Food and Drug Control (Beijing, China).

1.2 Sample preparation

YQSD was prepared as described in the text. 1.5mL of the decoction was diluted to 5mL with methanol. Then the solution was shaken well and centrifuged for 10 min at 12 000 r · min^-1^. The supernatant was filtered using a 0.22μm microporous filter membrane, which is the test solution.

1.3 Chromatographic conditions

UHPLC was performed using a C18 column ACQUITY UPLC® HSS T3 column (2.1×100 mm, 1.8 μm), a detection at variable wavelength (0~15min, 283nm; 15~22.75min, 250nm; 22.75~35min, 283nm). Mobile phase A was composed of 0.05% formic acid aqueous solution, and mobile phase B was composed of 100% acetonitrile. Injection volume was 2 μL. The gradient elution procedure was as follows, 0~3min, 95%~90%A; 3~5min, 90%~88%A; 5~7min, 88%~86%A; 7~15min, 86%~84%A; 15~20min, 84%~82%A; 20~22min, 82%~80%A; 22~26min, 80%~70%A; 26~29min, 70%~68%A; 29~31min, 68%~50%A; 31~33min, 50%~0%A; 33~34min, 0%~95%A; 34~35min, 95%~95%A. The volume flow rate was 0.2mL/min and column temperature was at 35 °C.

2. Results


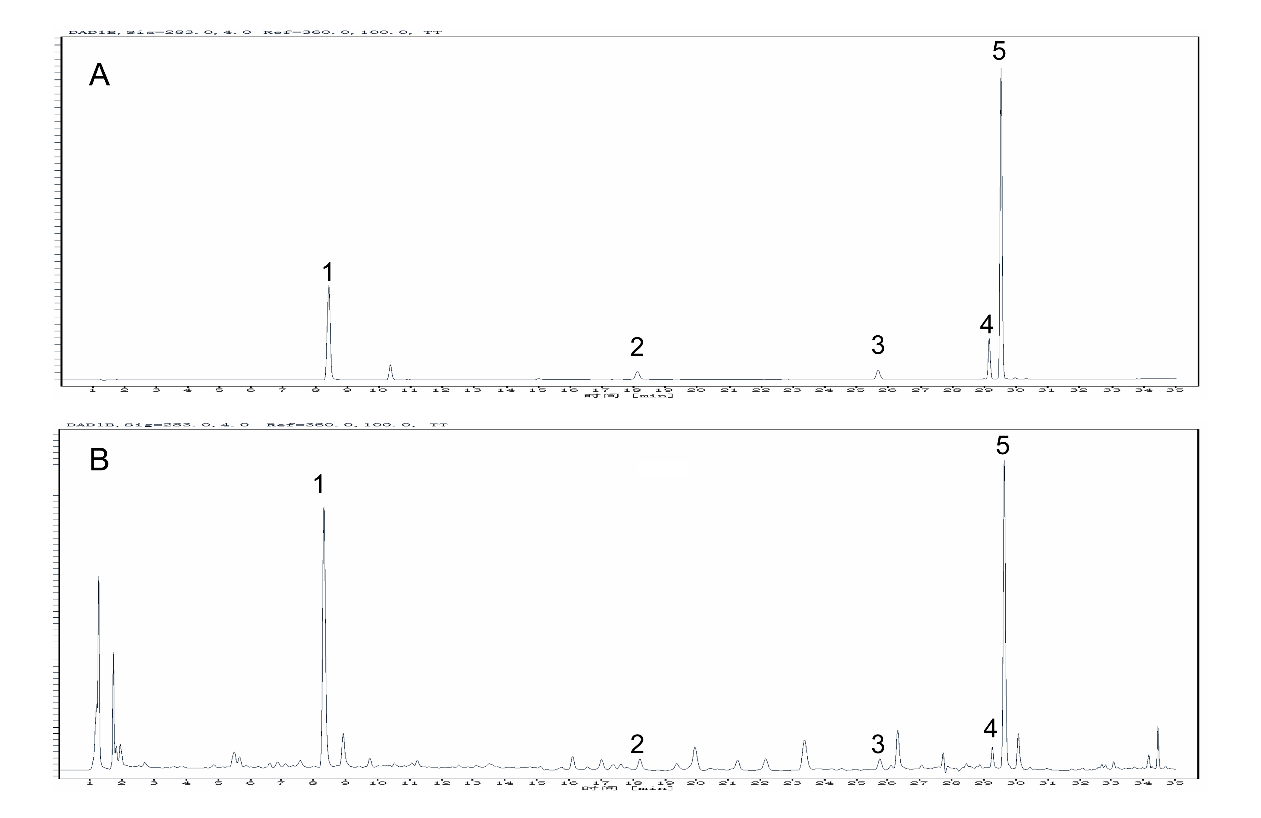
UHPLC showed peaks of chlorogenic acid, rutin, hesperidin, phillyrin, arctiin in both mixed standards and YQSD samples (Fig. S1 A and B). These substances in YQSD exhibited peak times consistent with the control standard at 8.29, 18.20, 25.71, 29.24, and 29.61 min, respectively. This identification confirmed the presence of chlorogenic acid, rutin, hesperidin, phillyrin, and arctiin in YQSD utilized in the present experiment and their contents was 118.99, 5.32, 3.34, 7.7, and 136.9 μg·mL^-1^, respectively.

Fig. S5 UHPLC analysis of Yinqiaosan decoction (YQSD) and standards. 1. chlorogenic acid, 2. rutin, 3. hesperidin, 4. phillyrin, 5. arctiin (A) Chromatogram of five mixed standards. (B) Chromatogram of YQSD.
