# Supplementary material for: The therapeutic effect of Yinqiaosan decoction against influenza A virus infection by regulating T cell receptor signaling pathway
Source: Heliyon. 2024 Aug 13;10(16):e36178. doi: 10.1016/j.heliyon.2024.e36178 (PMC11382312; doi:10.1016/j.heliyon.2024.e36178)
Supplement: Multimedia component 5 [file mmc5.docx]

Figure S3A-p-ZAP 70 Figure S3B- ZAP 70



Figure S3C-p-PI3K Figure S3D-PI3K

Figure S3E-GAPDH

Figure S3 Originals blots corresponding to Fig7C.
